# Identity matters - perceptions of inter-professional feedback in the context of workplace-based assessment in Diabetology training: a qualitative study

**DOI:** 10.1186/s12909-020-1932-0

**Published:** 2020-02-03

**Authors:** Katrin Feller, Christoph Berendonk

**Affiliations:** 1 Department of Diabetes, Endocrinology, Nutritional Medicine and Metabolism, Inselspital, Bern University Hospital, University of Bern, Bern, Switzerland; 2 Institute for Medical Education, University of Bern, Bern, Switzerland

**Keywords:** Postgraduate medical education, Formative assessment, Workplace-based assessment, Interprofessional feedback, Interprofessional education, Social identity

## Abstract

**Background:**

Inter-professional collaboration is acknowledged as essential for quality patient-care. However, little is known about receptiveness to inter-professional feedback in the postgraduate training. This study explores, in light of social identity theory, the perceptions of residents, supervising physicians and allied health care professionals regarding inter-professional feedback in the context of workplace-based assessment.

**Methods:**

For 6 months, residents in Diabetology at the University Hospital of Bern performed formative workplace-based assessments under direct observation of a supervising physician and an allied health care professional. Feedback from both observers was given to the resident after every assessment. Subsequently, focus group discussions were conducted to collect the participants’ perceptions of inter- and intra-professional feedback. Transcripts were analyzed qualitatively using a thematic analysis approach.

**Results:**

We identified four main themes: (1) Identity and hierarchy; (2) Interdependence of feedback source and feedback content; (3) Impact on collaboration and patient-care; (4) Logistical and organizational requirements. While different social identities are the source of inter-professional hierarchies, they did not impede the receptiveness to feedback. Perceived trustworthiness of the feedback was attributed with more importance than professional affiliations, whereas intra-professional hierarchies between physicians led to the perception of a more summative nature of the feedback and rather impeded receptiveness. According to the participants, inter-professional feedback raised awareness of the working reality of other team members and had a positive impact on communication between the different professional groups. Moreover, participants reported positive response from patients regarding the inter-professional collaboration they experienced. Considerable organizational effort is required to enable the parallel observation of a resident’s consultation by a supervising physician and an allied health care professional.

**Conclusions:**

Feedback from allied health care professionals can be a valuable learning resource for residents, given its role outside the sometimes conflicting area of intra-professional hierarchies. Inter-professional feedback in the context of workplace-based assessment carries the potential to strengthen collaboration between the different professional groups.

## Background

Inter-professional collaboration is acknowledged as a key competency, and is essential in order to provide safe, high-quality, patient-centered care [1, 2]. According to the CanMEDS Framework, inter-professional collaboration involves sharing knowledge and the willingness to learn together, which requires an understanding of the roles of others [3]. Physicians are expected to be receptive to the feedback of other health care members and to be able to manage differences in opinion. While inter-professional feedback is recognized as one important part of inter-professional teamwork, little is known about receptiveness to inter-professional feedback in general. One recent study found that students have positive perceptions of inter-professional feedback, without systematic bias against any specific professional group [4]. It seems that this receptiveness to inter-professional feedback is decreasing when medical trainees progress and eventually become residents [5, 6]. Residents report limited exposure to inter-professional feedback in clinical routine but would in principle see opportunities for an effective use of inter-professional feedback [5].

Social identity theory provides a powerful framework for illuminating inter-professional collaboration in medical education [7]. Social identity is defined as an individual’s self-concept based upon his or her perceived membership in a social group [8]. The theory conceptualizes social identity as a way in which to explain intergroup behaviors. One basic finding of social identity theory is that group membership is associated with positive attitudes towards in-group members and negative attitudes towards out-groups, and the theory predicts that hierarchies and stereotypes might limit acceptance of inter-professional feedback [9].

The present study focuses on the postgraduate level, in the subspecialty of Diabetology. Diabetes care is inherently inter-professional in nature, requiring a patient-centered team approach encompassing physicians, diabetes nurses, nutritionists and psychologists. All of these health care professionals work in a similar setting, providing one-to-one consultations with the patient, often caring for the same patient. While the focus of these consultations might differ depending on the professional affiliation, overlapping competencies among the health care professionals is a hallmark of diabetes care. Residents working in this subspecialty usually have approximately 4–5 years of work experience. As social identity theory predicts that achievement of professional identity is related to factors such as knowledge and practical experience [7], it can thus be assumed that the residents participating in the study have attained a firm professional identity as physicians.

Formalized inter-professional feedback is most often conducted within the format of multi-source feedback (MSF) [10], in which several co-workers anonymously rate a trainee with whom they have worked over a prolonged period of time according to predefined generic competencies. The interaction between trainee and co-workers is limited in this type of feedback, since trainees review the collated MSF with their supervisor and not with the individual feedback deliverers. Research in higher education has highlighted that anonymous marking might undermine the learning potential of feedback, as it deemphasizes the relationship between feedback deliverer and feedback receiver [11]. In workplace-based assessments like the Mini-CEX (Mini-Clinical Evaluation Exercise), a health care professional directly observes a trainee during an interaction with a patient, immediately followed by brief, structured feedback [12, 13]. In our study, we combined the aspect of direct interaction between trainee and observer with an added inter-professional dimension, using the format of inter-professional Mini-CEX. While workplace-based assessment tools like the Mini-CEX are less known in the context of inter-professional feedback, the feasibility of inter-professional workplace-based assessment has been demonstrated [14].

The aim of this study was to unravel the appraisal of inter-professional feedback in the setting of postgraduate training in light of social identity theory. More specifically, we were interested in the perceptions of residents, supervising physicians and allied health care professionals in the field of diabetology regarding receiving and delivering feedback in the context of workplace-based assessment.

## Methods

As the purpose of our study was of explorative nature and the source of data was the naturalistic setting a qualitative approach was adopted [15]. We pursued a social-constructivist perspective in order to explore participants’ views on their professional identity as well as their perception of giving and receiving feedback in the context of newly introduced inter-professional workplace-based assessment.

### Background

This study was conducted at the Department of Diabetology at the University Hospital of Bern. Participants were the entire team of physicians, consisting of seven residents and seven supervising physicians, as well as all nine members of the team of allied health care professionals (AHPs), consisting of four diabetes nurses, three nutritionists and two psychologists. The residents working in this subspecialty training possess at least 4 years of work experience, mostly in internal medicine, and some have previously worked as supervising physicians in general internal medicine. Being part of an outpatient clinic, all health care professionals perform one-to-one consultations with patients suffering from diabetes mellitus, providing advice regarding overlapping topics such as diabetes and nutrition, exercise, car driving, pregnancy etc., antidiabetic therapy (medications and insulin, pump instructions), adherence, as well as motivation and psychological support in facing the burden of a chronic disease.

In postgraduate training every resident in Switzerland must undergo four workplace-based assessments per year [16], in which a supervising physician observes a resident-patient interaction and subsequently provides structured feedback. These assessments have a formative purpose and are designed to promote learning. For the purpose of the present study, all residents were asked to perform their workplace-based assessments with an additional observer from the team of AHPs.

### Procedure

The study was divided into three phases:

1. Preparation phase
  In Spring/Summer 2017 inter-professional education sessions took place. Sessions were held on a regular basis (once per week), and were conducted and attended by all of the health care professionals. Contents were diabetes-related as well as medical education topics, the latter focusing on ‘how to provide feedback’.
2. Administration of inter-professional workplace-based assessments
  From September 2017 to February 2018, each of the seven residents in the Department of Diabetology were asked to undergo four inter-professional workplace-based assessments under direct observation of a supervising physician and an AHP. The residents were instructed to ask for specific feedback, and the observers were instructed to provide feedback which targeted behavior and contained suggestions for improvement. The purpose of the assessment was formative, with no summative elements. After every assessment, both observers provided the resident with written and oral feedback, simultaneously if possible but sometimes successively for organizational reasons. Of the seven residents, one resident underwent five inter-professional workplace-based assessments, three residents underwent four, two residents underwent three and one resident underwent two. Overall, a total of 25 inter-professional workplace-based assessments took place from September 2017 to February 2018.
3. Data collection
  Focus group discussions were held with all participants to collect data regarding delivering and receiving feedback in the context of workplace-based assessment.

We chose focus groups in order to seek a broad spectrum of views and to facilitate discussion through interaction between participants. Three focus group interviews were conducted from February to March 2018: one with the residents (six of the total of seven residents participated), one with the supervising physicians (all of the seven supervising physicians participated), and one with the AHPs (all of the four diabetes nurses, two of three nutritionists, and two of two psychologists participated). The focus group discussions took place with the two authors as facilitators and lasted for approximately 60 min. A semi-structured approach was underpinned by open-ended questions designed to elicit participants’ ideas and concepts. Examples of probing questions were: How was it for you to give feedback to a resident together with a supervising physician? (question to AHP); How was it for you to receive feedback about your work from a person who is not a physician? (question to residents); To what extent did the feedback from an AHP influence your feedback? (question to supervising physicians). The detailed interview guide is shown in the Additional file 1. Questioning evolved according to the participants’ responses. The discussions were audio-recorded and transcribed verbatim.

### Data analysis

We adopted a thematic analysis approach [17]. By analyzing the experiences and perspectives of the participants described in the transcripts, we aimed to identify factors that aid or impede the receptiveness to feedback and the willingness to give feedback, and to explore how this feedback influenced the collaboration between the three groups. The two authors independently coded all transcripts inductively. Social identity theory was used as sensitizing concept. Using constant comparative analysis, akin to grounded theory analytic techniques, codes were discussed and refined and emerging themes were developed iteratively [18].

### Research team

The study group comprised two researchers. KF is a specialist in internal medicine and in diabetology and endocrinology and is highly engaged in medical education. CB is a physician and senior medical educator who has extensive experience in the implementation of workplace-based assessment methods in under- und postgraduate education as well as experience in qualitative research.

### Ethical approval

All participants were informed about the goals and objectives of the study. Participation in the study was voluntary and participants could discontinue the study at any time. No incentive was offered. All participants signed an informed consent form. Confidentiality of data was maintained; data were only discussed within the research team. The study was deemed exempt from formal ethical approval based on institutional regulations, as no patient data was involved.

## Results

We identified four main themes: (1) Identity and hierarchy; (2) Interdependence of feedback source and feedback content; (3) Impact of inter-professional feedback on collaboration and patient-care; (4) Logistical and organizational requirements for implementing inter-professional feedback on an institutional level.

1. Identity and hierarchy: Multidimensionality of social identity, inter- and intra-professional hierarchies.

Residents participating in the study maintained a positive attitude towards feedback from allied health care professionals (AHPs). Even though there was a clear awareness of existing inter-professional hierarchies, residents’ professional identity as “physicians” did not impede their receptiveness to feedback from “non-physicians”. However, receiving and delivering inter-professional feedback was perceived as unusual and new:

*R5 (resident): Before performing an inter-professional workplace-based assessment, I was really wondering what this would feel like, to receive feedback from someone who is not a physician. Because, you know, there are some kinds of hierarchies in the hospital. And usually, it is us, the physicians, who tell them [the AHPs] how to work with the patients, and they ask us for our advice, even though, in fact, we are not their supervisors, of course.*

*AHP6: Yes, it is very unusual for us to give feedback to a physician. I have never done this before. There have been hierarchies in the hospital for decades, you know, and we all feel these hierarchies, even though we have good relationships with the physicians.*

Despite the apparent presence of this inter-professional hierarchy, it did not have a negative effect on residents’ perception of feedback from AHPs. On the contrary, the inter-professional feedback actually allowed an exchange and refection on observed performance with a focus on promoting learning.

*R5: It [the feedback] was totally on a level playing field, it was not strange and I did not feel criticized in any way. It was very constructive feedback. I was astonished about this; I thought it would be different.*

While interacting with the supervising physicians, the shared professional identity as physicians became less important and the identities of “trainee” and “supervisor” became more accentuated. These identities and associated intra-professional hierarchies led to the perception of a more summative nature of the workplace-based assessment, a fact that rather impeded receptiveness to feedback.

*R5: I caught myself selecting the supervising physicians. I chose those with whom I feel I have a good relationship. I told myself ‘let’s not expose yourself too much in this situation’, you know, so I consciously selected my medical supervisors. My performance could play a role in my annual evaluation, even though it should not in the context of this project, but you cannot exclude it completely.*

Supervising physicians also believed that the intended formative assessment might have felt more summative to the residents due to the perception of intra-professional hierarchies, making the residents choose “easy tasks” in order to perform well.

*S7 (supervising physician): I really do think that hierarchies played a role. I observed that residents chose tasks that were very easy for them, and I was wondering why this happened. Did they perceive this as a “school test situation”, where they will be judged?*

When the supervising physicians, together with an AHP, gave structured feedback to a resident about a directly observed performance, they became more aware of their identity as teachers.

*S4: In these inter-professional workplace-based assessments, I did learn a lot about our residents, but I also learned a lot about myself, about how I am doing as a teacher.*

(2) Interdependence of feedback source and feedback content: The role of the perceived trustworthiness of the feedback source and the task on which feedback is given.

While maintaining a positive attitude towards inter-professional feedback in general, not all feedback would have been perceived as trustworthy from the outset. Perceived trustworthiness of the feedback source, combined with the task on which the feedback was provided, played a crucial role in the residents’ receptiveness to inter-professional feedback.

*R2: If, for example, the nutrition specialist would have advised me on how to diagnose a hypercortisolism, I would have thought.. well, okay … But I think this is really related to the topics and tasks on which they give you the feedback. I think this plays the major role, and that’s why it was so appropriate, helpful and congruent for me when I received their [the AHPs’] feedback.*

Residents showed a greater openness to learning from other professionals if they perceived them to be competent in their domain and trustworthy. The focus of the feedback differed between supervising physicians and AHPs, reflecting what each group considered as important in their own profession. All residents agreed that feedback on communication skills is a specific competency of AHPs, and they showed high receptiveness to such inter-professional feedback.

*R1: I had this patient with type 1 diabetes. He had heard our advice at least a hundred times and still hadn’t achieved good glycemic control. After the assessment, the psychologist discussed different strategies for how to communicate with this patient. I found this feedback very helpful.*

A perceived strength of AHPs’ feedback was that it complemented the physicians’ feedback. Physicians and AHPs agreed that diabetology is inherently inter-professional in nature, and saw overlapping as well as complementary competencies as resources in inter-professional feedback.

*AHP2: I [AHP] was more focused on the consulting aspect, and she [supervising physician] added the medical input, it was very complementary and a pleasant cooperation.*

*S5: When it was about how to instruct a patient about hypoglycemia, the inputs of the diabetes nurse were extremely helpful. We are not used to explaining this to a patient in such a structured and clear manner. I realized that the resident was very satisfied with this feedback from an AHP.*

(3) The impact of inter-professional feedback.

Two subthemes emerged from this main theme: the impact of interprofessional feedback on (A) collaboration and (B) patient-care.

(A) The impact of inter-professional feedback on collaboration.

Many of the study participants noted an impact on inter-professional collaboration. For example, AHPs explained that being present at a physician’s consultation helped them to better understand the work of the residents.

*AHP2: It was interesting to see how physicians work in their consultations, and to realize all the challenges they are facing, working with demanding patients, with translators and so on, and working with very limited time resources.*

This inter-professional feedback not only fostered awareness of other team members but also actually had a positive impact on communication and collaboration between the different professions.

*AHP2: In the past, I always used to hesitate … the physicians are so busy, and I thought I should not to bother them, or I was wondering when would be the best time to ask them a question. And now, I just drop in and ask: Do you have a minute? … I also experienced some situations in which the residents actively contacted me about a patient and asked me if I could just drop by. So in my view, there is a lot more exchange and communication now between the professions.*

The inter-professional workplace-based assessments also promoted new inter-professional education sessions. Ideas for new contents within inter-professional education sessions arose during workplace-based assessments and were later implemented, such as a teaching session on functional intensified insulin therapy together with nutrition specialists and physicians.

(B) The impact of inter-professional feedback on patient-care.

Study participants felt that the inter-professional feedback was beneficial with regard to patient-care. AHPs described that being present at a resident’s consultation allowed them to complement their own consultations with the same patient later on, consolidating topics that had previously arisen in the physician’s consultation and expanding on them based on their own specialty.

*AHP7: I was lucky to be part of an inter-professional assessment and then, afterwards, to have the same patient in my consultation. This was very helpful, I could tie in with existing knowledge and experiences.*

Moreover, after only 4 months of conducting inter-professional workplace-based assessments, the effect of the improved collaboration was even noticeable for patients. Several study participants received positive feedback from patients regarding the inter-professional collaboration within the team.

*AHP6: Finally, patients feel that we are working together as a team. Just this morning, I met a patient who told me “You have an excellent collaboration here among physicians and nutrition specialists!” Moreover, I think he felt much safer, knowing that we are all moving in the same direction. Therefore, I really believe that not only we, but all the patients, benefit from this improved inter-professional collaboration.*

(4) Logistical and organizational requirements for implementing inter-professional feedback on an institutional level.

While perceiving inter-professional feedback as helpful and as promoting inter-professional collaboration, the study participants also mentioned the high degree of organizational effort required to enable the parallel observation of a resident’s consultation by a supervising physician and an AHP. All study participants expressed the need for institutional support for inter-professional workplace-based assessments.

When organizing inter-professional feedback, it is also important to consider the needs of all of the participants. In this project, AHPs felt that the organization was too focused on the residents’ agenda.

*AHP6: I had the impression that the organization was too oriented to the physician’s needs. This made it difficult for the other professions, because we are organized in a very different way.*

Given the organizational effort necessary for conducting inter-professional workplace-based assessments, institutional support in order to integrate inter-professional workplace-based assessments into daily work routine is highly desirable.

## Discussion

Our findings suggest that the social identity of all participants was affected when participating in an inter-professional workplace-based assessment (wpba). Residents seemed to assume different identities depending on whom they were interacting with in wpba. When they were conducting a wpba and were receiving feedback from a supervising physician, residents at times felt pushed into the role of a ‘subordinate’. The assumption of such an identity altered residents’ conception of formative wpba, giving it a more summative character. This perception was not particularly helpful in order to fully benefit from supervisors feedback and somehow contrasted with the perception of supervising physicians. Supervising physicians became more aware of their identity as teachers when they, together with AHP, were observing residents and giving structured feedback in the context of wpba. When interacting with AHP, residents seemed to embrace an identity as members of an (inter-) professional health care team and they viewed AHP as in-group members of this team. The feedback was thus received and accepted with much less anxiety. In parallel, the opportunity to participate in an inter-professional wpba made AHP feel a valuable part of that very team. At first glance, our results seem to contradict those of the literature [5, 6]. It should be noted, however, that the perception of feedback usefulness in most studies was at the meta-level and did not refer to a concrete interaction between professional groups, as is the case in this study. It seems that the concrete interaction within the framework of the wpba exerts a decisive influence on the perception of feedback usefulness and the identity of the persons involved. In the intra-professional constellation between supervising physicians and residents, the two groups became more aware of their differences, whereas in the inter-professional constellation between AHP and residents the similarities came to the fore.

Inter-professional feedback was valued as helpful and credible as long as the feedback source was considered trustworthy and competent in the respective domain. The importance of aligning feedback source and feedback content has been supported by numerous studies [19, 20]. There was a stable difference in the feedback content of AHPs relative to that of physicians, with the former focusing especially on communication skills and interaction with patients and the latter focusing on medical knowledge. This pattern has been reported by others [14]. Feedback content from AHP and supervising physician exerts an additive effect, sheds light on performance from different perspectives, and enables a more complete picture to be gained. The difference in focus reflected what each feedback provider deemed to be valuable and important within their own profession. This clear perception of the distinguished competencies rendered AHPs a credible feedback source, and fostered residents’ receptiveness to the inter-professional feedback.

Inter-professional collaboration is essential for high-quality patient-care [21]. Based on our sample, it seems that inter-professional feedback in the context of workplace-based assessment can promote collaboration. Protected time for inter-professional feedback led to a better understanding of one another and to an improved dialogue between the professions. Moreover, this inter-professional feedback appeared to act as a vehicle through which individuals could learn from each other. This resonates nicely with the notion of ‘communities of practice’, best described as ‘people who share a concern or a passion for something they do and learn how to do it better as they interact regularly’ [22] as well as with Carless´ findings that ‘trust’ and ‘dialogic feedback’ are key elements in learning [23].

Restraints of daily clinical practice (especially limited time) hinder a more profound exchange of knowledge, ideas and feedback among professionals on a regular basis. Inter-professional feedback in the context of workplace-based assessments need to be allocated protected time in order to be integrated into daily work routine. This requires substantial organizational and logistical efforts. To achieve sustainable implementation, all of the study participants expressed their wish for institutional support, recognizing the time and human resources required and the need to plan in advance accordingly. The rewards for the necessary institutional investment might be improved teamwork and better patient -care.

## Limitations

Some important limitations of the present study need to be mentioned. First, the study was conducted at one clinic and the number of participants and of the resulting inter-professional workplace-based assessments was small. Second, the study was conducted in a highly specific clinical context, within postgraduate subspecialty training in Diabetology, a specialty that is inherently inter-professional in nature. Positive attitudes towards inter-professional feedback might also reflect the positive climate within this specific team. Moreover, the character of the study is not merely explorative: The inter-professional educational sessions and the introduction of inter-professional workplace-based assessments are interventions that might have contributed to the positive perceptions towards inter-professional feedback. As a further limitation, the small number of participants reduced the possibility to anonymize the data, although data confidentiality was guaranteed. An awareness of the limited possibility of anonymity may have led to a modification of the statements and ideas emerging within the focus group interviews, especially when talking about sensitive issues like inter- and intra-professional hierarchies. One of the authors of the study was an interviewer in the focus group interviews as well as a member of the team of supervising physicians. To which extent this might have influenced / biased the results, must be left open. However, participating residents and AHP were quite willing to give critical feedback, this primarily concerned the role, and function of the supervising physicians respectively the physicians dominated agenda. Considering these aspects the topic of interest deserves further study.

The present study also has some specific strengths. First, the relationships between identity and feedback that emerged from analysis have theoretical explanation and seem to be of a fundamental nature, rather than representing a specific phenomenon of diabetology. Accordingly, it is plausible that the topic of identity and feedback and its interrelationships can also be demonstrated in other disciplines with other occupational groups. Second, our findings may help to get a better grasp about strengths, opportunities, dangers and weaknesses of workplace-based assessment at large - a topic of essential importance for competency based education. Third, the fact that patients also noticed an improved collaboration between the different professional groups appears to be an indicator of the potential that seems to be inherent in formalized inter-professional feedback. This finding is particularly impressive given the limited intervention and short duration of our project.

In our study, feedback was given in a unidirectional manner, from supervising physicians and AHPs to residents. The perception of mutual, bidirectional feedback among all health care workers remains to be investigated. While the perception of inter-professional feedback was positive in our study, its impact on performance improvement also warrants further research.

## Conclusions

Residents in subspecialty training in Diabetology maintain a positive attitude towards feedback from allied health care professionals in the context of workplace-based assessment. Inter-professional feedback might be a powerful resource for learning through formative assessments, given its role outside the sometimes conflicting area of an intra-professional hierarchy among residents and supervising physicians. Credibility of the feedback source, in combination with the task on which the feedback was provided, is the single most important factor affecting feedback receptiveness. Inter-professional workplace-based assessments require institutional support in order to be performed on a regular basis, but carry the potential to improve inter-professional collaboration.

## Supplementary information

**Additional file 1:** Interview Guide

## Data Availability

Datasets analysed during this study are available from the corresponding author on reasonable request.

## Abbreviations

AHP: Allied health care professional
Mini-CEX: Mini-clinical evaluation exercise
Wpba: Workplace-based assessment
