# Supplementary material for: Identity matters - perceptions of inter-professional feedback in the context of workplace-based assessment in Diabetology training: a qualitative study
Source: BMC Med Educ. 2020 Feb 3;20:33. doi: 10.1186/s12909-020-1932-0 (PMC6998209; doi:10.1186/s12909-020-1932-0)
Supplement: Supplementary file 1 — Additional file 1: Interview Guide [file 12909_2020_1932_MOESM1_ESM.docx]

**Interview guide**

**Introduction**

The literature shows that interprofessional teamwork leads to improved patient care. Interprofessional cooperation also includes interprofessional feedback. We already know from other studies that interprofessional feedback in the context of workplace-based assessments is feasible. However, little is known about the extent to which feedback in continuing medical education is accepted and can be implemented across professional boundaries. We are interested in your experiences with interprofessional feedback in the context of workplace-based assessments in the past five months. So, there are no "right or wrong" answers, but we are interested in your opinion, your concrete examples and lived experiences with interprofessional feedback.

**Questions to allied health care professionals**

- What was it like for you as non-physician to look over the shoulders of the residents in their work and give them feedback?
- Do you remember a situation in which your feedback seemed particularly useful / valuable to the resident?
- Were there any situations where it was difficult for you to give feedback to the resident?
- How was your feedback received by the residents?
- Were there any situations where the residents did not agree with your feedback?
- What was it like for you to give feedback to a resident together with a supervising physician?
- Did the feedback from the supervising physician influence your opinion or feedback?
- Were there any situations where your feedback was very different from the one from the supervising physician?
- Was there a situation where you did not agree with the feedback from the supervising physician?
- Based on the experiences in the present project, has your interaction with the physicians changed in the clinical routine?

**Questions to supervising physicians**

- Giving the feedback, you were- at least partly- the three of you together. I would be interested to know how this feedback process was carried out by the three of you?
- Can you describe an example in which the allied health care professional (AHP) gave the resident a particularly good / helpful feedback?
- Can you describe an example in which the AHP gave the resident a feedback with which you did not agree / which you found questionable?
- To what extent did the feedback of the AHP influence your feedback?
- To what extent will the experience with the interprofessional feedback influence the interaction with the other professional groups in regular clinical routine?
- Can you imagine an AHP looking over your shoulder during a consultation and giving you feedback afterwards?

**Questions to residents**

- Can you describe an example where the allied health care professional (AHP) gave you a particularly good / helpful feedback?
- Can you give an example of a particularly helpful feedback from a supervising physician?
- Was there a situation where you did not agree with the feedback from the AHP?
- Was there a situation where you did not agree with the feedback from the supervising physician?
- What is it like to accept a feedback from someone who is not a physician?
- Do you remember a situation where the feedback from the AHP and the feedback from the supervising physician was different, or even contradictory?
- How did you choose the two people (supervising physician and AHP) giving feedback?
- Do you have the impression that the interprofessional workplace-based assessments have influenced your cooperation with AHPs?
